# Expanding Disease Spectrum Associated With Puerperal Mastitis

**DOI:** 10.1155/S1064744997000689

**Published:** 1997

**Authors:** Gregg L. McAdoo, Gilles R. G. Monif

**Affiliations:** Department of Obstetrics and Gynecology Creighton University School of Medicine 601 North 30th Street Omaha NE 68131 USA

## Abstract

*Background: Staphylococcus aureus* and the β-hemolytic streptococci are the commonest causes of puerperal mastitis which tends to be a localized disease process. This report describes 2 cases attributable to these bacteria that resulted in extramammary involvement and augmented morbidity.

*Cases: *Two cases of postpartum mastitis are described, one leading to necrotizing fasciitis caused by group A streptococci and the other leading to toxic shock syndrome (TSS) caused by *S. aureus*.

*Conclusion: *The spectrum of disease commonly attributed to mastitis occurring in this setting should be expanded.


Infectious Diseases in Obstetrics and Gynecology 5:376–379 (1997)
© 1998 Wiley-Liss, Inc.

### Expanding Disease Spectrum Associated With Puerperal Mastitis

Gregg L. McAdoo and Gilles R.G. Monif*
*Department of Obstetrics and Gynecology, Creighton University School of Medicine, Omaha, NE*

ABSTRACT

*Background:* Staphylococcus aureus and the β-hemolytic streptococci are the commonest causes of puerperal mastitis which tends to be a localized disease process. This report describes 2 cases attributable to these bacteria that resulted in extramammary involvement and augmented morbidity.
*Cases:* Two cases of postpartum mastitis are described, one leading to necrotizing fasciitis caused by group A streptococci and the other leading to toxic shock syndrome (TSS) caused by *S. aureus*.
*Conclusion:* The spectrum of disease commonly attributed to mastitis occurring in this setting should be expanded. Infect. Dis. Obstet. Gynecol. 5:376–379, 1997. © 1998 Wiley-Liss, Inc.

KEY WORDS
bacterial infection; necrotizing fasciitis; toxic shock syndrome; *Staphylococcus aureus;* group A streptococci

The group A streptococcus (*Streptococcus pyogenes*) and *Staphylococcus aureus* are common causes of puerperal mastitis. In recent times, the streptococcal and staphylococcal toxic shock syndrome (TSS) and the sensationalization of so-called "flesh-eating" bacteria have revived broad public interest in these bacteria.[1–12] Infection of the breast caused by these bacteria is still usually thought of in terms of simple mastitis responsive to penicillinase-resistant, first-generation penicillins. The purpose of this article is to reiterate the expanded spectrum of disease caused by these common breast pathogens.

### CASE 1

The patient was an 18-year-old Hispanic female, $G_1P_1$, who had had a spontaneous vaginal delivery 18 days prior to her admission. Approximately 3 days prior to admission, she began experiencing chills, fever, and intense pain in her right breast. The pain became progressively worse until the day of her admission, at which time she experienced a definite decrease in its intensity. The baby refused to take milk from that breast.

On the patient's admission, her vital signs were a temperature of 39.83°C (103.7°F), pulse of 160 beats/min, blood pressure of 88/40 mm, and respirations of 18/min. The pertinent physical findings were limited to an erythematous, indurated right breast which was grossly distorted because of edema. The nipple was swollen 3–4 times its normal size. Thick, yellowish, white milk was present at the nipple. The patient was treated with intravenous (IV) nafcillin, 2 g q 4 h. An examination of the breast the next morning revealed 2 distinct areas of violaceous skin. The first of these lesions was 6 x 7 cm in maximum diameter adjacent to the areola (Fig. 1). The second lesion was 6 × 10 cm, involving the lateral aspect of the right breast (Fig. 2). Within the first lesion was an area of black skin which measured 1 x 3 cm. Adjacent to this area was a small bleb filled with serosanguinous fluid. An ultrasonographic evaluation of the breast demonstrated small-to-moderate pockets of interstitial

*Correspondence to: Dr. Gilles R.G. Monif, Department of Obstetrics and Gynecology, Creighton University School of Medicine, 601 North 30th Street, Omaha, NE 68131.

Received 9 July 1996

Accepted 27 September 1996

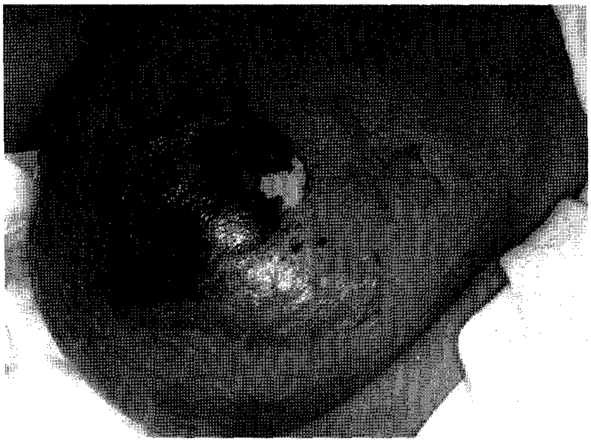

Fig. 1. Violaceous areas with focal necrosis within an erythematous swollen breast infected with group A streptococci.

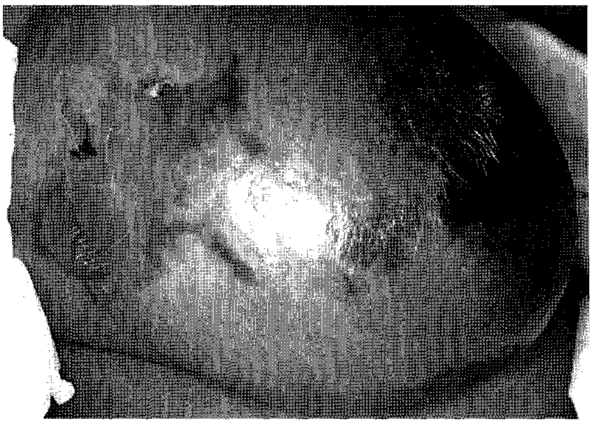

Fig. 2. Lateral view of breast in Figure 1. Note the detachment of the superficial epidermis and presence of fluid-filled bleb. Figures 1 and 2 were taken 6 h after the diagnosis of necrotizing fasciitis. During that time, focal necrosis and obvious cutaneous gangrene had developed.

fluid comparable with cellulitis within the interstitial septae. The diagnosis of necrotizing fasciitis caused by group A streptococcus was made on clinical grounds. The subsequent tissue and breast milk samples all grew group A streptococci. The nafcillin was discontinued. She was placed on ceftriaxone, 2 g q 12 h, and gentamicin 2 mg/kg qd, before being transferred to the general surgical service 17 h after admission.

The surgeons managing this case chose to see what could be achieved with maximum antibiotic therapy. On the 4th hospital day, she was taken to the operating room, at which time 3 blocks of skin tissue measuring 9 x 8 x 2.5 cm, 12 x 14 x 4 cm, and 10 x 11 x 2 cm were removed. The histologic examination revealed marked acute cellulitis edema, tissue necroses, and focal abscess formation. A brown stain of the tissue samples revealed gram-positive cocci. Because of continued fever and signs of tissue necrosis at the operative site, the patient was taken back to the operating room on the 3rd postoperative day (7th hospital day), at which time 3-cm incisions were made, 15 cm apart, extending from 6 cm below the right breast along the right auxiliary line to approximately 2 cm above the iliac crest. On the 9th hospital day, she again was taken to the operating room for additional debridement. At this time, an aggressive wound resection was carried out until a very good blood return emanated from all incision sites. Fourteen days following her admission, she was taken back to the operating room for the 4th time, at which time a large (30 cm in maximum diameter) tissue-flap reconstruction was carried out. She was discharged from the hospital 5 days later.

### CASE 2

The patient was a 26-year-old white female who presented to the emergency room 8 days postpartum following a normal spontaneous vaginal delivery. She reported the symptoms of fever, nausea, vomiting, diarrhea, and mild soreness in her left breast of 2 days duration. Her physical examination on admission revealed a temperature of 38.72°C (101.7°F), with orthostatic blood-pressure changes. The patient had left breast tenderness associated with localized erythema and induration. The right breast revealed generalized tenderness and fullness. The remainder of the examination was unremarkable. Her laboratory values on admission showed a WBC count of 4,500/mm$^3$ (45% bands), platelets of 195,000/mm$^3$, and blood urea nitrogen (BUN)/creatinine (CR) of 10/.8. A diagnosis of postpartum mastitis was made. She was treated with IV nafcillin, 2 g q 4 h, and electrolyte replacement. On the 2nd hospital day, she developed a diffuse watery diarrhea and sunburn-like rash involving her entire face, thorax, and abdomen. Her temperature was 40°C (104°F) and her WBC count was 17,200/mm$^3$ (62% bands). The BUN and CR had increased to 28 and 1.7, respectively. Her diagnosis was changed to TSS attributable to postpartum staphylococcal mastitis. The nafcillin was discontinued and IV vancomycin therapy was initiated. Her fluid and electrolyte balances were aggressively managed. A fecal *Clostridium difficile* toxin assay, blood cultures, chest X-ray, abdominal ultrasound, and ultrasound of the breasts were all negative. A culture of the breast drainage grew a pure culture of *S. aureus* which was sensitive to vancomycin. The patient's temperature and laboratory values returned to normal over the next 32 h. She was discharged home on hospital day 5.

### DISCUSSION

Necrotizing fasciitis initially was termed "streptococcal gangrene" by Meleney.[1] The disease was subsequently renamed necrotizing fasciitis in order to reinforce the concept characteristic of this type of infection that, while progressively destroying fascia and fat, the infection spares the skin and muscle from the progression of cutaneous erythematous edema to pink and then blue areas of coloration in association with blisters and blebs.[2,3] A failure to perform the appropriate surgical debridement is associated with exaggerated morbidity or mortality, as illustrated in the case reported.[4] The change in the character of the pain, characteristic of this disease, is attributable to skin infection, which, as such, indicates the need for aggressive surgical debridement.

Progressively more and more cases of staphylococcal TSS are occurring in non-menstruating women. In obstetrical patients, TSS has been described following vaginal deliveries, cesarean deliveries, and spontaneous and therapeutic abortions.[5–10] Of the 130 cases of non-menstrual TSS reported by Reingold et al.,[10] one was associated with mastitis.[10] As illustrated, an early diagnosis and aggressive intervention correlate well with good outcomes.

TSS is a multisystem illness. A syndrome consisting of malaise, myalgia, low-grade fever, nausea, and vomiting or diarrhea may antecede the overt disease. In the full-blown, acute systemic illness, the patient presents with fever of >38.9°C (102°F), sore throat, headache, chills, severe hypotension, myalgia, pharyngitis, conjunctivitis, leukocytosis, and generalized arthralgia. The rash is usually a consistent part of the syndrome, presenting as a diffuse, sunburn-like, blanching macular erythema.

In contrast to the cases reported in the American literature, TSS associated with puerperal mastitis is a reasonably well-delineated entity in the European literature.[9–12] de Save et al.[11] identified 2 cases in reviewing Great Britain's experience with TSS. Isolated cases have been reported from both Sweden and The Netherlands.[9,12] Demey et al.[9] reported a case of TSS due to postpartum staphylococcal mastitis that progressed to abscess formation. The patient was treated with plasma expanders until hemodynamic stability was achieved; IV cloxacillin, 8 g q d; and 1 dose of 500 mg of amikacin. Echography and CT scans of the right breast were negative for abscess formation. The patient was discharged from intensive care after 7 days, at which time the right breast appeared normal. A CT scan performed 3 weeks after her hospital admission revealed a well-delineated, fluid-filled cavity deep in the right breast. *S. aureus* was again cultured, but the patient was clinically well, with only localized fluctuation unassociated with any signs of inflammation or TSS. The absence of TSS despite large staphylococcal abscesses fits well with recently published *in vitro* data which show that TSST-1 production is maximized during the linear growth of *S. aureus* under anaerobic growth conditions while toxin production is diminished significantly.

The key to effective therapy in both conditions is an awareness of the entities and an understanding of the events that combine to produce these disease entities. Severe, invasive group A streptococcal breast disease is not well documented in the literature. As illustrated in our case, even when the entity is accurately diagnosed early in its course, a failure to understand its pathogenesis can lead to extensive loss of skin and subcutaneous tissues.

### REFERENCES

1. Meleney FL: Hemolytic *Streptococcus* gangrene. Arch Surg 9:317–364, 1924.
2. Schwartz B, Facklam RR, Brieman RF: Changing epidemiology of group A streptococcal infection in the USA. Lancet 336:1167–1171, 1990.
3. Stevens DL: Invasive group A streptococcus infections. Clin Infect Dis 14:2–13, 1992.
4. Stevens DL, Bryant AE, Yan S: Invasive group A streptococcal infection: New concepts in antibiotic treatment. Int J Antimicrob Agents 4:297–301, 1994.
5. Green SL, LaPeter KS: Evidence for postpartum toxic shock syndrome in a mother-infant pair. Am J Med 72: 169, 1982.
6. Whitfield JW, Valenti WM, Magnussen CR: Toxic shock in the puerperium. JAMA 246:1806–1807, 1981.

7. Bracero L, Bowe E: Postpartum toxic shock syndrome. Am J Obstet Gynecol 143:478–479, 1982.
8. Chow AW, Wittman BK, Bartless BA, et al.: Variant postpartum toxic shock syndrome with probable postpartum transmission to the neonate. Am J Obstet Gynecol 148:1074–1079, 1984.
9. Demey HE, Hautekeete ML, Buytaert P, et al.: Mastitis and toxic shock syndrome. Acta Obstet Gynecol Scand 68:87–88, 1989.
10. Reingold AL, Hargrett NT, Dan BB: Non-menstrual toxic shock syndrome: A review of 130 cases. Ann Intern Med 96:871–874, 1982.
11. de Save MJ, Hawtin P, Wiencke AA: Toxic shock syndrome in Britain: Epidemiology and microbiology. Postgrad Med J 61(Suppl 1):5–21, 1985.
12. Niessan GJ, Bartels CC, Degener JE, Stibbe J: Mastitis and toxic shock syndrome. Neth J Surg 37:101–104, 1985.
